# Supplementary material for: Mume Fructus reduces interleukin-1 beta-induced cartilage degradation via MAPK downregulation in rat articular chondrocytes
Source: PLoS One. 2024 May 8;19(5):e0302906. doi: 10.1371/journal.pone.0302906 (PMC11078424; doi:10.1371/journal.pone.0302906)

Fig 4. Effect of Mume Fructus (MF) on the protein levels of metalloproteinase-3 (MMP3), metalloproteinase-13 (MMP13), a disintegrin and metalloproteinase with thrombospondin motifs 5 (ADAMTS5), and collagen type II alpha 1 chain (COL2A1) in rat articular chondrocytes.

Figure 4. A

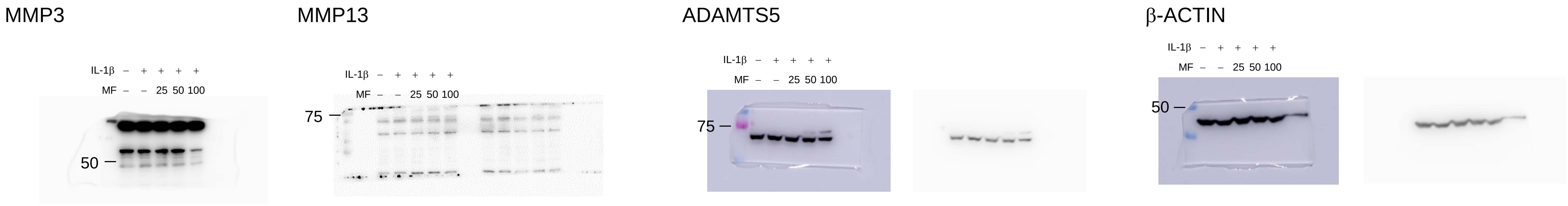

Figure 4. B

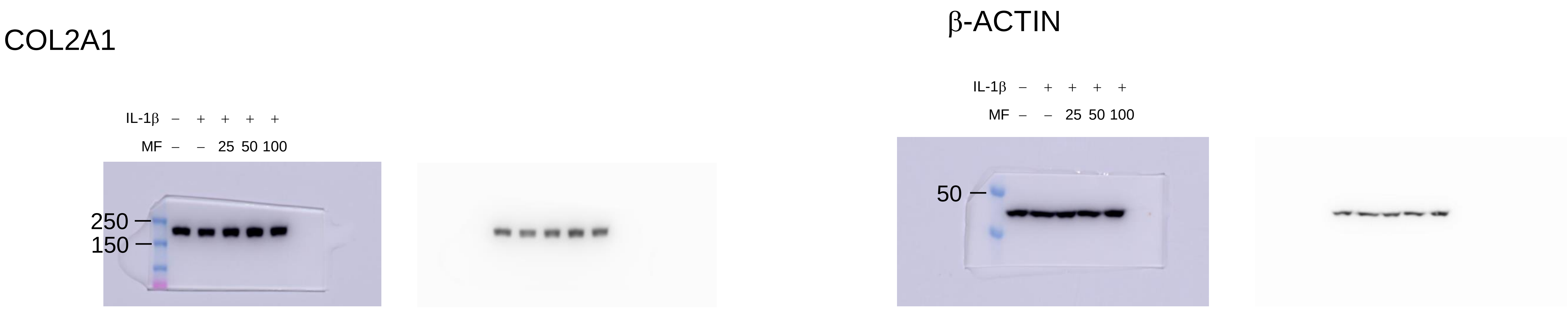

Fig 7. Inhibition of inflammatory response upon Mume Fructus (MF) treatment via mitogen-activated protein kinase–nuclear factor-kappa B (MAPK–NF-κB) signaling in rat articular chondrocytes.

Figure 7. A

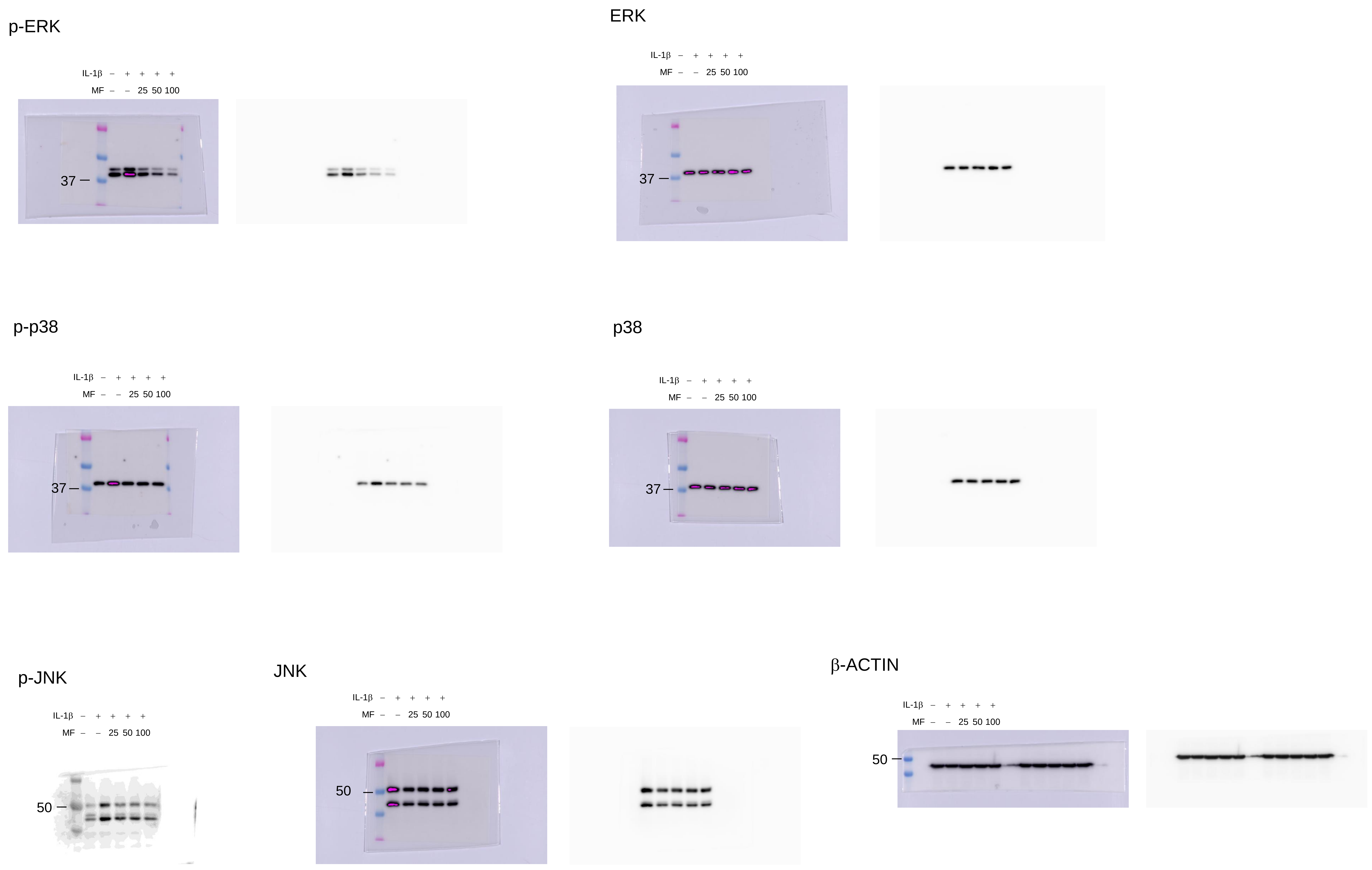

Figure 7. B

p-p65

IL-1β – + + + +

MF – – 25 50 100

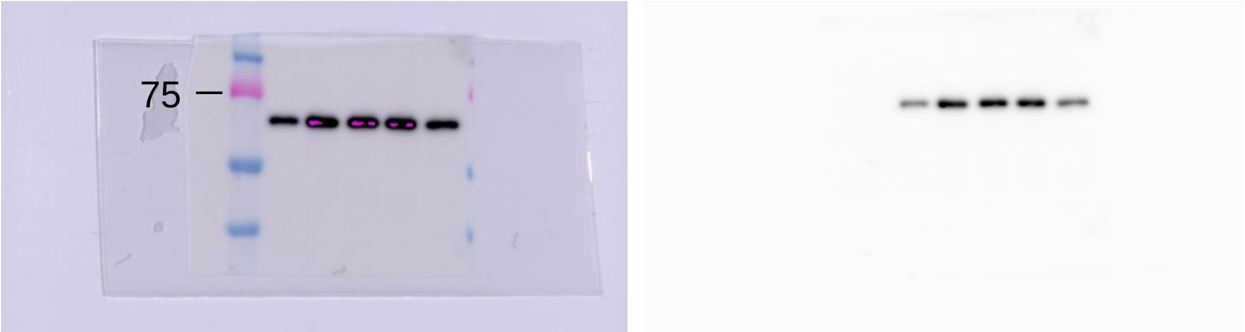

p65

IL-1β – + + + +

MF – – 25 50 100

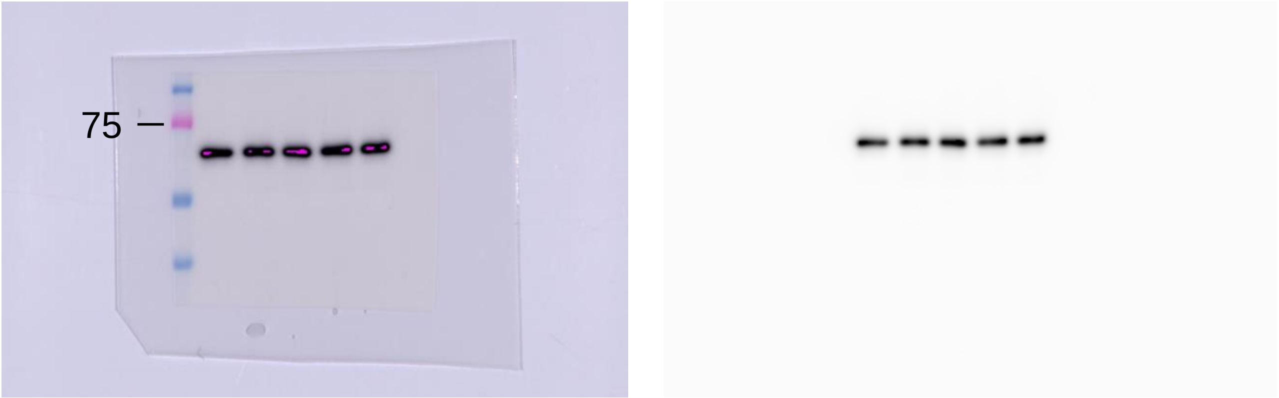

β-ACTIN

IL-1β – + + + +

MF – – 25 50 100

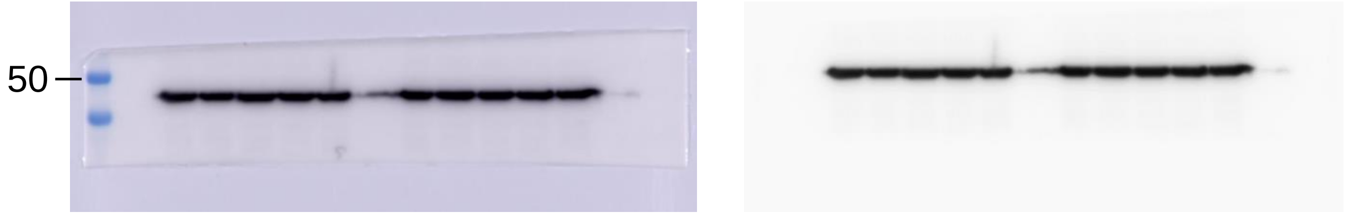

Figure 7. C

COX-2

IL-1β – + + + +

MF – – 25 50 100

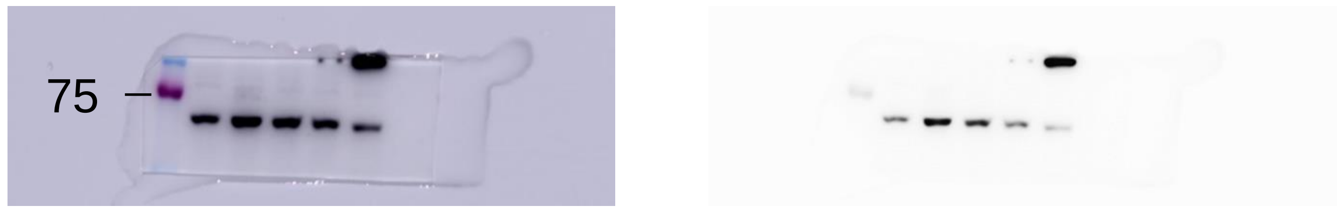

β-ACTIN

IL-1β – + + + +

MF – – 25 50 100

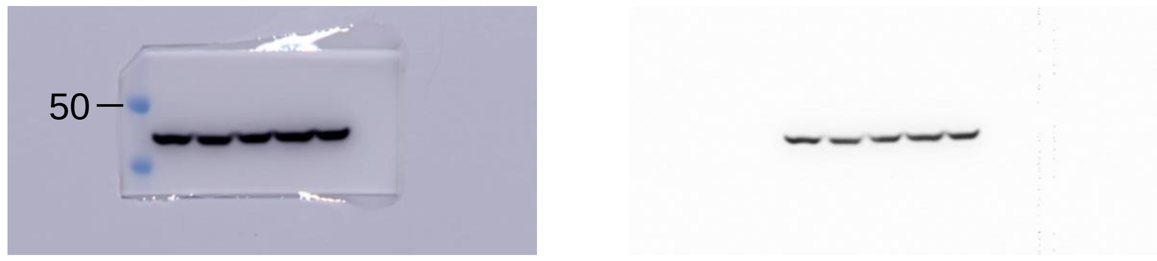

Supplement: S1 File — (PDF) [file pone.0302906.s006.pdf]
