# Supplementary material for: Mume Fructus reduces interleukin-1 beta-induced cartilage degradation via MAPK downregulation in rat articular chondrocytes
Source: PLoS One. 2024 May 8;19(5):e0302906. doi: 10.1371/journal.pone.0302906 (PMC11078424; doi:10.1371/journal.pone.0302906)
Supplement: Attachment — Submitted filename: 231126 PLOSE ONE Mume Fructus Review.docx [file pone.0302906.s008.docx]

**PLOSE ONE**

Manuscript number:	*PONE-D-23-27950*

Article Type:	*Research article*

**Mume Fructus reduces interleukin-1 beta-induced cartilage degradation via MAPK downregulation in rat articular chondrocytes**

Doo Ri Park, Chanhwang Yeo, Jee Eun Yoon, Eun Young Hong, Bo Ram Choi, Seung Ho Baek, Yoon Jae Lee, **In-Hyuk Ha**

Short title:	*MF reduces cartilage degradation via MAPK signalling*

Keywords:	*Articular cartilage; Osteoarthritis; Chondrocytes; Cartilage inflammation; Mume*

*fructus; herbal medicine*

**Summary of the research article**

In this interesting article, Park and coworkers focus their attention on possible anti-inflammatory benefits of *Mume fructus* (MF), a fumigated derivative of the *Prunus mume* (PM) fruit. Indeed, on cartilage and macrophage cell models, the authors observed that MF leads to metalloproteinases (MMPs) reduction and accumulation of collagen type II and aggrecan in interleukin-1β (IL-1β) in rat chondrocytes through down-regulation of MAPK and NF-κB signalling. In parallel, MF may have additional beneficial effects on reduction of LPS-induced inflammation in macrophages. It was also noted that specifically MF and not its initial product PM to possess such anti-inflammatory benefits on IL-1β-derived inflammatory signalling in rat chondrocytes. Last but not least, the authors determined the aqueous composition of both PM and MF via high-performance liquid chromatography (HPLC). This analysis allowed the authors to show a different 4-CQA content in MF compared to PM, which could raise hypotheses about its possible role in the anti-inflammatory events observed in aforementioned experiments.

Although the study highlighted undeniable relationships between MF and the anti-inflammatory power of the latter in rat chondrocytes, some additional experiments could bring greater value to the study. Furthermore, although there are publications in this regard, the point developed by the authors regarding side effects of NSAIDs that may not be present in the natural active ingredient of MF has not been comprehensively demonstrated in this article. Consequently, this article must be enriched with new experiments and resubmitted for approval. Below I will indicate the points to be developed to enrich the story on MF.

**Major points**:

The authors correctly introduced the concept of possible pleiotropic effects of NSAIDs on chondrocytes. A publication by *Chang et al.* (Toxicology, 2006) analyzed the possible effects on proliferation and cell death of different groups of NSAIDs in cultured epiphyseal-articular chondrocytes of fetal rats. The authors concluded that non-selective NSAIDs cause reduced thymidine incorporation at dose-dependent concentrations, cell cycle arrest in the G_0_/G_1_ phase, and induction of both apoptosis and cell necrosis. Cyclooxygenase-2 (COX-2) selective NSAIDs accentuate these effects on chondrocytes in a less marked way and DFU, an analog of rofecoxib, has no effect on both cell proliferation and necrosis/apoptosis. Although the aforementioned research article shows the effects of NSAIDs on chondrocytes, a further experiment comparing NSAIDs and MF is important to understand whether MF may possess similar pleiotropic effects. A cytotoxicity test should be performed with NSAIDs (both non-selective and COX-2 selective NSAIDs since they have different power effects) at high concentrations as a positive control (DFU could be used as a negative control) and compared with MF at different doses to establish a possible non-involvement or even a benefit of MF versus cytotoxicity. I would suggest to add [3H]-thymidine incorporation assay, TUNEL stain (terminal deoxy-nucleotidyl transferase mediated dUTP nick end labeling stain), cell cycle kinetics by flow cytometry and caspase 3 activity assay to determine the effects of MF on cell proliferation, cell cycle and apoptosis/necrosis. I would apply the following experiments for both cultured rat condrocytes and macrophages to observe whether MF could have non-toxic effects on both chondrocytes and macrophages.

In figure 4, the authors performed a western blot analysis to observe the role of IL-1β signaling in inflammation. It would be interesting to also introduce known inhibitors (U0126 for ERK1/2, SB239063 for p38 and SP600125 for JNK) in order to compare the inhibitory effect with MF.

In figure 6, an immunofluorescence analysis of rat chondrocytes shows that the intensity of PM is comparable to the IL-1β group, suggesting no type of modulation of IL-1β signaling by PM. In addition to immunofluorescence, the authors could add a western blot with relative expression analysis of p-ERK/total ERK, p-p38/total p38 and p-JNK/total JNK to confirm at a biochemical level the absence of signaling modulation IL-1β versus MF (always consider cells treated with IL-1β only as a positive control).

Figure 7 of the article by Park and coworker shows an HPLC analysis between MF and PM with the aim of dissecting the profile of the chemical composition of the latter in order to understand why MF has an effect on the signaling dictated by IL-1β , while this is not the case for PM. The authors observed higher 4-CQA abundance in MF compared to PM, suggesting this molecule as responsible for the anti-inflammatory nature shown by MF. However, a molecular analysis of this molecule in the modulation of IL-1β-associated signaling remains to be determined/confirmed. Cryptochlorogenic acid powder can be purchased and can be applied in some experiments. Western blot analyses and immunofluorescence of rat chondrocytes treated with IL-1β, MF, PM and 4-CQA can be performed to re-observe the gene and protein expression of MMPs and collagen/aggrecan, as well as observe the phosphorylation levels of p-ERK, p-p38 and p-JNK, to confirm the key role of 4-CQA in inflammatory modulation in chondrocytes.

**Minor points**

*Discussion section (lines 331-337)*. I would reccomend to add the following citation :

Chang JK, Wu SC, Wang GJ, Cho MH, Ho ML. Effects of non-steroidal anti-inflammatory drugs on cell proliferation and death in cultured epiphyseal-articular chondrocytes of fetal rats. Toxicology. 2006 Dec 7;228(2-3):111-23. doi: 10.1016/j.tox.2006.08.028. Epub 2006 Aug 30. PMID: 17045721.

*Figure section*. Improve figures quality. I opened the document several times and the file lost its initial resolution. I would suggest saving the images in TIFF format and mounting them in Adobe® Photoshop/Illustrator to preserve resolution quality.

*Figure section*. Add concentration units in each graph.

*Figure section.* Quantitative analyzes of Western Blots show no error bars. I assume the experiments were not repeated. Repeat the experiments in order to obtain the minimum number of biological replicates so that a given significance can be obtained (if a significant finding is observed).

*Figure 7*. The 3 molecules taken into account in the HPLC peak analysis are shown. Molecules have letters A to C, peaks numbers from 1 to 3. It is true that it is specified in the text under image 7, but in my opinion it is easier to choose either numbers or letters to visually appreciate which molecule corresponds to which peak.
