# Supplementary material for: Mume Fructus reduces interleukin-1 beta-induced cartilage degradation via MAPK downregulation in rat articular chondrocytes
Source: PLoS One. 2024 May 8;19(5):e0302906. doi: 10.1371/journal.pone.0302906 (PMC11078424; doi:10.1371/journal.pone.0302906)
Supplement: Attachment — Submitted filename: Response to reviewers.doc [file pone.0302906.s009.doc]

**Reviewer #1**

*1. The authors concluded that MF inhibited interleukin-1 beta-induced cartilage degradation via MAPK down regulation, citing references 17–19. They also mentioned that IL1β induces its destructive effects through activation of the MAPK signaling pathway. More proof, though, is required to support this. This theory might be validated by gene silencing certain MAPK signaling pathway components.*

**Response**: We thank you for your comment. First, we thank the reviewer for the valuable suggestions regarding our experiments on the mechanism involving the MAPK signaling pathways. We conducted the study based on the view that verifying the efficacy of *MF* in inhibiting IL1β-induced inflammatory markers or cartilage degradation in rat chondrocytes through experiments was the most appropriate method of evaluating the efficacy of MF. The main objective of our research was to explore the function of IL-1β signaling in inflammatory processes as it pertains to our study. Although existing inhibitors might offer insightful data regarding the implicated pathways, we deliberately concentrated on the innate reaction to MF, avoiding the influence of external inhibitors. By adopting this method, we were able to scrutinize the inherent signaling pathways and the natural effects of MF on chondrocytes.

*2. What software has been utilized for the analysis of immunofluorescence and western blot images?*

**Response**: We apologize for not providing more detailed information on the analytical methods. We added more description about the method of data analysis under Materials and Methods (Page 9, lines 168-172; Page 10, lines 190-191)

The protein bands were visualized using an Amersham Imager 600 imaging system (GE Healthcare Life Sciences, Uppsala, Sweden) and an enhanced chemiluminescence (ECL) system (Bio-Rad, Hercules, CA, USA). The protein levels were quantified using ImageJ (NIH, Bethesda, Maryland, USA).

The fluorescence intensity was analyzed using ImageJ (NIH, Bethesda, Maryland, USA).

*3. Fig. 3 (B&C) are the same whether LPS treatment is applied or not. They are combinable.*

**Response**: We thank you for your comment. Following the suggestion, we revised the manuscript by combining the experimental results in RAW264.7cells in Figure 6. We address this issue in the following text added to the Result (Page 26, lines 513-517).

To assess the effect of MF on NO production in RAW 264.7 cells, we examined the inflammation induced by LPS and multiple doses of MF extract for over 24 h. MF treatment inhibited NO production in RAW 264.7 cells in a dose-dependent manner with and without LPS stimulation (Fig 6B). Moreover, TNF-α, IL-6, and IL-1β levels significantly increased in LPS-treated cells and decreased in MF-treated cells (Fig 6C).

**Fig 6. Effects of Mume Fructus (MF) on lipopolysaccharide (LPS)-induced inflammatory responses in RAW 264.7 cells.** RAW 264.7 cells were treated with or without MF extract (25, 50, and 100 μg/mL) and exposed to LPS (1 μg/mL) for 24 h. (A) Cell viability was assessed using the CCK-8 assay (n=4). (B) Nitric oxide (NO) production was determined using an NO assay (n=4). (C) mRNA levels of tumor necrosis factor-alpha (TNF-α), interleukin 6 (IL-6), and interleukin 1 beta (IL-1β) were detected via quantitative reverse transcription-PCR (n=4). Data are expressed as mean ± standard deviation (SD). One-way ANOVA was performed followed by Dunnett’s multiple comparison test. *P < 0.05, **P < 0.01, ***P < 0.001 vs. LPS-treated group. #P < 0.05, ##P < 0.01, ###P < 0.001 vs. control group.

*4. Table 2 should include a description of the abbreviations for the phosphorylated forms of NF-ΚB, p65, ERK, JNK, and P38.*

**Response**: We thank you for your comment. We have made the necessary revisions and added the relevant sentence. We address this issue in the following text added to the Materials and methods (Page 10, lines 174-179).

MMP3, metalloproteinase-3; MMP13, metalloproteinase-13; ADAMTS5, a disintegrin and metalloproteinase with thrombospondin motifs 5; COL2A1, collagen type II alpha 1 chain; NFκB, nuclear factor kappa B; p-NFκB, phohphorylated nuclear factor kappa B; ERK, extracellular signal-regulated kinase; p-ERK, phohphorylated extracellular signal-regulated kinase; JNK, c-Jun N-terminal kinase; p-JNK, phohphorylated c-Jun N-terminal kinase; p-P38, phohphorylated P38; COX-2, cyclooxygenase-2

*5. Verify the chromogram again in Fig. 7-D. There is no greater abundance of the 4-CQA peak in MF extract than in PM.*

**Response**: We apologize for causing confusion as the description of the results and graphical illustration were not in agreement due to the mix-up in the notations of Mume Fructus (MF) and *Prunus mume* (PM) in the existing HPLC chromatograms. We now present the newly revised HPLC chromatograms in Fig 3 and detailed information on the retention times, area, etc. for HPLC peaks of Mume fructus (MF) and *Prunus mume* (PM) extracts in Table 5. We address this issue in the following text added to the Results (Page 23, lines 455-470).

HPLC chromatograms showed peaks of neochlorogenic acid (Fig 3A), chlorogenic acid (Fig 3B), and cryptochlorogenic acid (4-CQA, Fig 3C) in both MF and PM extracts. Unlike the peak area distribution of the PM extract, the peak area of 4-CQA in the MF extract was more than 4 times larger than that in the PM extract; the peak area ratio in the MF extract was 22.62% whereas that in the PM extract was 5.42%, indicating a marked difference between the two peak areas. These results suggest that the 4-CQA component in MF inhibits OA signaling in the primary chondrocytes of rats. These results suggest that the 4-CQA component in MF inhibits OA signaling in the primary chondrocytes of rats.

**Fig 3.** **Chemical structure of the three main ingredients of** **Mume Fructus (MF) and** ***Prunus mume* (PM) extracts, (A) neochlorogenic acid (B) chlorogenic acid, and (C) cryptochlorogenic acid.** **HPLC chromatograms of (D) MF, and (E) PM extracts.** mAU refers to the absorbance unit.

**Table 5** Retention times and area peak of Mume fructus (MF) and *Prunus mume* (PM) extract (n=4).

| Name | | Retention time | Area | Area % | Height | Height % |
| --- | --- | --- | --- | --- | --- | --- |
| Neochlorogenic acid | | 10.82 | 3416.25 | 78.56 | 261.84 | 85.00 |
|  |  | 10.90 | 3347.41 | 78.29 | 256.00 | 85.69 |
|  |  | 10.96 | 3382.73 | 79.72 | 287.15 | 81.72 |
|  |  | 10.87 | 3293.04 | 79.31 | 279.02 | 82.90 |
| Chlorogenic acid | | 13.85 | 3699.57 | 76.60 | 274.59 | 79.78 |
|  |  | 13.90 | 3625.57 | 76.53 | 267.78 | 79.86 |
|  |  | 13.85 | 3076.21 | 75.58 | 227.75 | 76.33 |
|  |  | 13.78 | 3558.78 | 76.29 | 263.18 | 76.93 |
| Cryptochlorogenic acid | | 14.93 | 5581.36 | 90.09 | 366.02 | 92.60 |
|  |  | 14.84 | 5580.47 | 92.84 | 368.36 | 93.38 |
|  |  | 14.74 | 5854.79 | 90.71 | 384.17 | 92.93 |
|  |  | 14.63 | 5547.67 | 90.97 | 362.62 | 91.94 |
| Mume Fructus | Neochlorogenic acid | 10.75 | 4194.30 | 37.70 | 248.85 | 34.68 |
|  |  | 10.76 | 4196.36 | 37.67 | 253.10 | 35.20 |
|  |  | 10.77 | 4150.94 | 36.93 | 253.46 | 34.93 |
|  |  | 10.87 | 4041.00 | 37.77 | 313.25 | 39.30 |
|  | Chlorogenic acid | 13.82 | 1690.92 | 15.20 | 127.88 | 17.82 |
|  |  | 13.82 | 1696.46 | 15.23 | 128.58 | 17.88 |
|  |  | 13.83 | 1697.50 | 15.10 | 128.83 | 17.75 |
|  |  | 13.91 | 1665.17 | 15.56 | 126.96 | 15.93 |
|  | Cryptochlorogenic acid | 14.80 | 2516.18 | 22.62 | 147.30 | 20.53 |
|  |  | 14.80 | 2524.91 | 22.67 | 148.34 | 20.63 |
|  |  | 14.81 | 2526.57 | 22.48 | 148.34 | 20.44 |
|  |  | 14.95 | 2406.64 | 22.50 | 150.63 | 18.90 |
| Prunus mume | Neochlorogenic acid | 10.72 | 7363.15 | 39.01 | 530.13 | 37.32 |
|  |  | 10.72 | 7363.08 | 38.71 | 529.47 | 37.25 |
|  |  | 10.81 | 7288.23 | 37.15 | 503.99 | 36.23 |
|  |  | 10.72 | 7381.71 | 39.32 | 527.79 | 37.33 |
|  | Chlorogenic acid | 13.83 | 2906.94 | 15.40 | 219.76 | 15.47 |
|  |  | 13.83 | 2904.89 | 15.27 | 219.57 | 15.45 |
|  |  | 13.90 | 2851.50 | 14.54 | 217.62 | 15.64 |
|  |  | 13.83 | 2908.66 | 15.50 | 219.70 | 15.54 |
|  | Cryptochlorogenic acid | 14.936 | 1022.46 | 5.42 | 68.90 | 4.85 |
|  |  | 14.933 | 1022.52 | 5.38 | 69.50 | 4.89 |
|  |  | 14.955 | 942.33 | 5.02 | 69.72 | 4.94 |
|  |  | 14.927 | 941.90 | 5.02 | 69.48 | 4.91 |

Area, area under the peak; Area %; percentage of the peak area in the entire chromatogram; Height, peak height; Height %, percentage of the peak height in the entire chromatogram.

*6. In Discussion, lines 346–348 "The current study revealed that…… chondrocytes," but we are unable to draw the conclusion that the primary cause of the decrease in MMP3/13-ADAMTS-5 is the accumulation of COL2A as a result of MF treatment of chondrocytes. While over-expression of COL2A1 may be a useful treatment strategy for osteoarthritis, it is unable to decrease matrix metalloproteinase enzyme expression.*

**Response**: We apologize for not providing sufficient description and causing difficulties in the reviewer’s understanding. We revised the sentence for a more straightforward understanding of the efficacy of MF. We address this issue in the following text added to the Discussion (Page 31, lines 617-619).

Our results verified the effects of MF treatment in inhibiting MMP3/13 and increasing COL2A1 expression, which were combined to demonstrate the efficacy of MF treatment in OA signaling inhibition (Fig 8).

*7. The authors found that the NFĸB and MAPK pathways are in charge of the OA cartilage destruction after studying the IL1β induced inflammatory signaling pathway in rat chondrocytes. Along these lines, a pertinent question concerning LPS-induced inflammation in macrophages emerges and needs to be addressed. Is there a comparable signaling pathway regarding the inflammation that LPS causes in macrophages?*

**Response**: We apologize for not providing sufficient description and causing difficulties in the reviewer’s understanding. We provided additional explanations regarding OA and inflammatory responses. We address this issue in the following text added to the Discussion (Page 30, lines 610-616).

The pathophysiology of OA is characterized by the breakdown of the ECM of articular cartilage by proteinases, whose expression is upregulated by inflammatory stimuli [61]. These inflammatory responses occur in chondrocytes and synovial macrophages [62]. Among the pathways involved in inflammatory responses, MAPKs and NF-кB are well known to play key roles [63]. Therefore, we examined and confirmed the anti-inflammatory effects of MF through gene expression of TNF-α, IL-6, and IL-1β in RAW264.7 cells and also in chondrocytes.

Reviewer #2:

*1. Metallopeptidases should be replaced with* *metalloproteinases throughout the manuscript.*

**Response**: We thank you for your comment. We have made the necessary revision under figure legend (Page 22, lines 440).

metalloproteinase-3 (MMP3)

*2. Line 64: Are there any references for the use of Jaseng Woongayoungsin-hwan in herniated discs and lower limb pain?*

**Response**: We thank you for your comment. We added more detailed information from the existing literature on the use of Jaseng Woongayoungsin-hwan. We address this issue in the following text added to the Introduction (Page 4, lines 75-82).

~~Studies have shown that Woongayoungsin-hwan reduces pain and improves life functions [27, 28].~~ For patients with a herniated intervertebral disc, herbal medicine treatment with Woongayoungsin-hwan has been proven effective in reducing low back pain, showing an improvement with a quick return to the activities of daily life activities and routine [27]. For patients with muscular atrophy in the lower limbs caused by a condition of peripheral neuropathy, Korean medicine treatment with Woongayoungsin-hwan has exhibited a significant therapeutic effect with a gradual increase in the duration of self-walking exercise time [28].

*3. Line 71: improves life functions needs to be rephrased. The same for the next sentence lines 72-74.*

**Response**: We thank you for your comment. We deleted the sentence including “*improves life functions”* and added more information from the existing literature on the use of Jaseng Woongayoungsin-hwan. We address this issue in the following text added to the Introduction (Page 4, lines 75-82).

~~Studies have shown that Woongayoungsin-hwan reduces pain and improves life functions [27, 28].~~ For patients with a herniated intervertebral disc, herbal medicine treatment with Woongayoungsin-hwan has been proven effective in reducing low back pain, showing an improvement with a quick return to the activities of daily life activities and routine [27]. For patients with muscular atrophy in the lower limbs caused by a condition of peripheral neuropathy, Korean medicine treatment with Woongayoungsin-hwan has exhibited a significant therapeutic effect with a gradual increase in the duration of self-walking exercise time [28].

*4. Since MF and PM are basically the same fruit, why both were tested?*

**Response**: We apologize for not providing sufficient description and causing difficulties in the reviewer’s understanding. MF and PM are both fruits of Chinese plum, but MF is obtained through a fumigation process by harvesting near-mature fruits. We revised with additional information to the text regarding the differences between MF and PM. We address this issue in the following text added to the Discussion (Page 29, lines 568-584).

PM, a herbal medicinal plant commonly used in traditional Korean medicine and folk remedies, is the fruit of the Chinese plum (*Prunus mume* Sieb. et Zucc.). It has been reported to exhibit antimicrobial activity [40], and is effective against gastric secretion in rats [41] and diabetes [42]. PM has different names and uses depending on the time of harvest and processing method, and it is generally classified as follows: Cheongmae is a green fruit with hard pulp and a strong sour and astringent taste; Cheongmae in steamed and dried state is called Geummae; Cheongmae pickled in brine and sun-dried is called Baekmae; Cheongmae with its pericarp removed and blackened to charcoal is called Omae (MF); and the yellow fruit with the ripe, fragrant smell is called Hwangmae [43]. MF is processed by removing the pericarp and pits of near-mature Cheongmae picked from mid-June to early July, dried, and steam baked to black in a straw fire. MF is widely used as a medicinal herb in traditional Korean and Chinese medicine [44]. Previous studies on the effects of MF have reported its antioxidant activity [45], antimicrobial effects against bacteria causing food poisoning/gastroenteritis [46], antitumor effects [47], and hypoglycemic effects [48]. However, few studies have specifically investigated the potential of using MF in the treatment of OA.

*5. GAPDH antibody is missing from Table 2.*

**Response**: We apologize for causing confusion by using two different housekeeping genes. Both ACTβ and GAPDH are commonly used as housekeeping genes, but due to internal circumstances in the authors’ laboratory, a supply of β-actin antibodies was not available, and we had to use GAPDH instead in the experiment. We performed replicate experiments and revised the results with those obtained from using ACTβ as housekeeping genes instead of the previous results with GAPDH.

*6. Why concentrations of MF ranging from 0 to 100 μg/mL were used? What is the evidence that the activity is efficient in this range?*

**Response**: We thank you for your comment. In previous studies using MF, concentrations widely ranging from 0.5, 2, 5, 10, 20, 30, 50, 70, 100, 150, 200, to 300ug/ml were used for experiments [1-4]. In addition, in experiments with chondrocytes using natural products, the concentration in the range of 0 - 100 μg/mL was mainly used; thus considering these ranges of concentration, the concentrations of MF in this study ranged from 0 to 100 μg/mL [5-7].

*7. According to Fig1A, MF treatment seems to induce cell proliferation. How was this taken into consideration for all the subsequent experiments?*

**Response**: The cell counting Kit-8, the method we used to perform cell viability assay, is a convenient method to detect cytotoxicity under various conditions or concentrations of samples. Cell viability was confirmed after the treatment of samples, and the counts represent the number of healthy cells. The fact that cell death did not occur in chondrocytes even after treatment with MF for more than 24 hours indicates that no toxicity of MF was confirmed in cell-level experiments. Therefore, the confirmed cell viability may be considered as an indicator that incubation with various doses of MF was performed without problems in chondrocytes and RAW264.7 cell.

*8. Why are there no statistical analysis in western blots? If the authors have not performed a statistical test, all statements for significant results need to be rephrased.*

**Response**: We thank you for your comment. For western blot analysis, we rechecked about replicate measurements, and revised the legends of the Figures and the graphs used. We address this issue in the following text added to the Results (Page 25, lines 490-498; Page 28, lines 542-555).

**Fig 4. Effect of Mume Fructus (MF) on the protein levels of metalloproteinase-3 (MMP3), metalloproteinase-13 (MMP13), a disintegrin and metalloproteinase with thrombospondin motifs 5 (ADAMTS5), and collagen type II alpha 1 chain (COL2A1) in rat articular chondrocytes.** Rat articular chondrocytes were treated with or without MF extract (25, 50, and 100 μg/mL) and exposed to IL-1β (10 ng/mL) for 30 h. (A) MMP3, MMP13, ADAMTS5, and (B) COL2A1 protein expression was assessed using western blot analysis (n=4). Data are expressed as mean ± standard deviation (SD). One-way ANOVA was performed followed by Dunnett’s multiple comparisons test. *P < 0.05, **P < 0.01, ***P < 0.001 vs. IL-1β-treated group. #P < 0.05, ##P < 0.01, ###P < 0.001 vs. control group.

**Fig 7. Inhibition of inflammatory response upon Mume Fructus (MF) treatment via mitogen-activated protein kinase–nuclear factor-kappa B (MAPK–NF-κB) signaling in rat articular chondrocytes.** Rat articular chondrocytes were treated with or without MF extract (25, 50, and 100 μg/mL) and exposed to IL-1β (10 ng/mL) for (A) 30 min, (B) 1 h, and (C, D) 30 h (n=4). (A) Protein expression of phohphorylated extracellular signal-regulated kinase (p-ERK), phohphorylated c-Jun N-terminal kinase (p-JNK), and phohphorylated P38 (p-p38) assessed via western blot analysis. (B) Protein expression of phohphorylated P65 (p-p65) assessed via western blot analysis. (C) Protein expression of cyclooxygenase-2 (COX-2) assessed via western blot analysis. (D) tumor necrosis factor-alpha (TNF-α), interleukin 6 (IL-6), and interleukin 1 beta (IL-1β) mRNA levels were detected using quantitative reverse transcription-PCR. Data are expressed as mean ± standard deviation (SD). One-way ANOVA was performed followed by Dunnett’s multiple comparisons test. *P < 0.05, **P < 0.01, ***P < 0.001 vs. IL-1β-treated group. #P < 0.05, ##P < 0.01, ###P < 0.001 vs. control group

*9. No information for the biological/technical replicates has been included in methods or in the legends.*

**Response:** We apologize for omitting the description of the replicate measurements, causing difficulties in understanding. We rechecked the number of replicate measurements, and revised the legends of the Figures and the Tables. We address this issue in the following text added to the Results (Page 21-22, lines 427-446; Page 23, lines 463-466; Page 25-26, lines 490-506; Page 27, lines 518-527; Page 28, lines 542-555).

**Fig 1. Comparison of the cytotoxicity and chondrogenic gene expression efficacy of Mume Fructus (MF) and *Prunus mume* (PM) on primary rat chondrocytes.** Rat articular chondrocytes were treated with or without MF and PM extracts (25, 50, and 100 μg/mL) and exposed to IL-1β (10 ng/ml) for 30 h. (A) Cell viability was assessed using the CCK-8 assay (n=4). (B, C) Metalloproteinase-3 (MMP3), metalloproteinase-13 (MMP13), a disintegrin and metalloproteinase with thrombospondin motifs 5 (ADAMTS5), collagen type II alpha 1 chain (COL2A1), and aggrecan (ACAN) mRNA levels were assessed using quantitative reverse transcription-PCR (n=5). Data are expressed as mean ± standard deviation (SD). One-way ANOVA was performed, followed by Dunnett’s multiple comparison test. *P < 0.05, **P < 0.01, ***P < 0.001 vs. IL-1β-treated group. #P < 0.05, ##P < 0.01, ###P < 0.001 vs. control group.

**Fig 2. *Prunus mume* (PM) had no effect on IL-1β-induced signaling in primary chondrocytes.** Expression of metalloproteinase-3 (MMP3) (A), a disintegrin and metalloproteinase with thrombospondin motifs 5 (ADAMTS5) (B), and collagen type II alpha 1 chain (COL2A1) (C) was evaluated using immunofluorescence staining (n=5). Scale bar = 25 μm. (D) The fluorescence intensity was quantified using image J. Data are expressed as mean ± standard deviation (SD). One-way ANOVA was performed, followed by Dunnett’s multiple comparison test. *P < 0.05, **P < 0.01, ***P < 0.001 vs. IL-1β-treated group. #P < 0.05, ##P < 0.01, ###P < 0.001 vs. control group.

**Table 5.** Retention times and area peak of Mume Fructus (MF) and *Prunus mume* (PM) extract (n=4).

**Fig 4. Effect of Mume Fructus (MF) on the protein levels of metalloproteinase-3 (MMP3), metalloproteinase-13 (MMP13), a disintegrin and metalloproteinase with thrombospondin motifs 5 (ADAMTS5), and collagen type II alpha 1 chain (COL2A1) in rat articular chondrocytes.** Rat articular chondrocytes were treated with or without MF extract (25, 50, and 100 μg/mL) and exposed to IL-1β (10 ng/mL) for 30 h. (A) MMP3, MMP13, ADAMTS5, and (B) COL2A1 protein expression was assessed using western blot analysis (n=4). Data are expressed as mean ± standard deviation (SD). One-way ANOVA was performed followed by Dunnett’s multiple comparisons test. *P < 0.05, **P < 0.01, ***P < 0.001 vs. IL-1β-treated group. #P < 0.05, ##P < 0.01, ###P < 0.001 vs. control group.

**Fig 5. Effects of** **Mume Fructus (MF) on IL-1β-induced signaling in primary chondrocytes. (A-C)** The expression of matrix metalloproteinase (MMP) 3 (A), a disintegrin and metalloproteinase with thrombospondin motifs 5 (ADAMTS5) (B), and collagen type II alpha 1 chain (COL2A1) (C) was evaluated using immunofluorescence staining (n=5). Scale bar = 25 μm. (D) Fluorescence intensity was quantified using Image J. Data are expressed as mean ± standard deviation (SD). One-way ANOVA was performed, followed by Dunnett’s multiple comparisons test. *P < 0.05, **P < 0.01, ***P < 0.001 vs. IL-1β-treated group. #P < 0.05, ##P < 0.01, ###P < 0.001 vs. control group.

**Fig 6. Effects of Mume Fructus (MF) on lipopolysaccharide (LPS)-induced inflammatory responses in RAW 264.7 cells.** RAW 264.7 cells were treated with or without MF extract (25, 50, and 100 μg/mL) and exposed to LPS (1 μg/mL) for 24 h. (A) Cell viability was assessed using the CCK-8 assay (n=4). (B) Nitric oxide (NO) production was determined using an NO assay (n=4). (C) mRNA levels of tumor necrosis factor-alpha (TNF-α), interleukin 6 (IL-6), and interleukin 1 beta (IL-1β) were detected via quantitative reverse transcription-PCR (n=4). Data are expressed as mean ± standard deviation (SD). One-way ANOVA was performed followed by Dunnett’s multiple comparison test. *P < 0.05, **P < 0.01, ***P < 0.001 vs. LPS-treated group. #P < 0.05, ##P < 0.01, ###P < 0.001 vs. control group.

**Fig 7. Inhibition of inflammatory response upon Mume Fructus (MF) treatment via mitogen-activated protein kinase–nuclear factor-kappa B (MAPK–NF-κB) signaling in rat articular chondrocytes.** Rat articular chondrocytes were treated with or without MF extract (25, 50, and 100 μg/mL) and exposed to IL-1β (10 ng/mL) for (A) 30 min, (B) 1 h, and (C, D) 30 h (n=4). (A) Protein expression of phohphorylated extracellular signal-regulated kinase (p-ERK), phohphorylated c-Jun N-terminal kinase (p-JNK), and phohphorylated P38 (p-p38) assessed via western blot analysis. (B) Protein expression of phohphorylated P65 (p-p65) assessed via western blot analysis. (C) Protein expression of cyclooxygenase-2 (COX-2) assessed via western blot analysis. (D) tumor necrosis factor-alpha (TNF-α), interleukin 6 (IL-6), and interleukin 1 beta (IL-1β) mRNA levels were detected using quantitative reverse transcription-PCR. Data are expressed as mean ± standard deviation (SD). One-way ANOVA was performed followed by Dunnett’s multiple comparisons test. *P < 0.05, **P < 0.01, ***P < 0.001 vs. IL-1β-treated group. #P < 0.05, ##P < 0.01, ###P < 0.001 vs. control group.

*10. The representative images and the quantification in Fig2C are not in agreement.*

**Response**: We apologize for causing difficulties in understanding due to the inconsistency between the representative images and the graph for quantification in the immunohistochemistry experiment of COL2A1 according to the treatment with different concentrations of MF in chondrocytes. After careful reexamination of the experimental data, we revised the graph accordingly.

*11. What is the reason for using GAPDH in some WB and ACTβ in other for normalisation?*

**Response**: We apologize for causing confusion by using two different housekeeping genes. Both ACTβ and GAPDH are commonly used as housekeeping genes, but due to internal circumstances in the authors’ laboratory, a supply of β-actin antibodies was not available, and we had to use GAPDH instead in the experiment. We performed replicate experiments and revised the results with those obtained from using ACTβ as housekeeping genes instead of the previous results with GAPDH.

*12. Since the authors aim to show the anti-inflammatory a=effects of MF in RAW 264.7 cells, Western blots for TNFα, IL6 and IL1β must be performed as well.*

**Response**: First of all, we thank the reviewer for the valuable suggestion. We acknowledge that the reviewer’s idea is helpful in improving the quality of our work to a higher level. We planned our study with the main objective of evaluating the efficacy of MF in chondrocytes. Our experiments confirmed that MF treatment inhibited inflammation in chondrocytes and that it was effective in suppressing inflammatory responses in RAW 264.7 cells. In this process, our experiments focused on markers related to inhibition of cartilage degradation rather than inflammation-related markers. Although the data presented in this study may seem to be lacking in some aspects to reviewers, please consider the fact that this is the first study to verify the efficacy of MF in chondrocytes.

*13. It seems that there is a repetition of results in Fig1 and Fig5 as well as Fig2 and Fig6 with regards to MF. Furthermore, it is not clear what were the concentrations used for MF or PM in Fig6. In addition, since the experiments were repeated, why are the fold changes in all 3 gene expressions different for MF?*

**Response**: We apologize for causing problems for reviewers in understanding this manuscript due to the poor organization and presentation of the experimental results of MF. The results of Fig 1 and Fig 5 in the previous manuscript were combined and organized into a new Fig 1, making the visual comparison of the efficacy of MF and PM on chondrocytes more intuitive. We changed the overall logical flow of the manuscript, we also changed the order of the figures in the existing manuscript. We expect that the newly revised manuscript will improve our understanding of the efficacy of MF in chondrocytes. We address this issue in the following text added to the Results (Page 21, lines 427-437).

**Fig 1. Comparison of the cytotoxicity and chondrogenic gene expression efficacy of Mume Fructus (MF) and *Prunus mume* (PM) on primary rat chondrocytes.** Rat articular chondrocytes were treated with or without MF and PM extracts (25, 50, and 100 μg/mL) and exposed to IL-1β (10 ng/ml) for 30 h. (A) Cell viability was assessed using the CCK-8 assay (n=4). (B, C) Metalloproteinase-3 (MMP3), metalloproteinase-13 (MMP13), a disintegrin and metalloproteinase with thrombospondin motifs 5 (ADAMTS5), collagen type II alpha 1 chain (COL2A1), and aggrecan (ACAN) mRNA levels were assessed using quantitative reverse transcription-PCR (n=5). Data are expressed as mean ± standard deviation (SD). One-way ANOVA was performed, followed by Dunnett’s multiple comparison test. *P < 0.05, **P < 0.01, ***P < 0.001 vs. IL-1β-treated group. #P < 0.05, ##P < 0.01, ###P < 0.001 vs. control group.

*14. The authors wrote that “the neochlorogenic and chlorogenic acid contents were similar in MF and PM extracts, but 4-CQA was more abundant in MF than in PM as per peak area value analysis”. Where is the quantification for this? This is not evident from Fig7D and the chromatograms should be presented equally, i.e. all baselines should start at 0 mAU.*

**Response**: We apologize for causing confusion as the description of the results and graphical illustration were not in agreement due to the mix-up in the notations of Mume Fructus (MF) and Prunus mume (PM) in the existing HPLC chromatograms. We now present the newly revised HPLC chromatograms in Fig 3 and detailed information on the retention times, area, etc. for HPLC peaks of Mume fructus (MF) and *Prunus mume* (PM) extracts in Table 5. We address this issue in the following text added to the Results (Page 23-24, lines 455-470).

HPLC chromatograms showed peaks of neochlorogenic acid (Fig 3A), chlorogenic acid (Fig 3B), and cryptochlorogenic acid (4-CQA, Fig 3C) in both MF and PM extracts. Unlike the peak area distribution of the PM extract, the peak area of 4-CQA in the MF extract was more than 4 times larger than that in the PM extract; the peak area ratio in the MF extract was 22.62% whereas that in the PM extract was 5.42%, indicating a marked difference between the two peak areas. These results suggest that the 4-CQA component in MF inhibits OA signaling in the primary chondrocytes of rats. These results suggest that the 4-CQA component in MF inhibits OA signaling in the primary chondrocytes of rats.

**Fig 3.** **Chemical structure of the three main ingredients of Mume Fructus (MF) and *Prunus mume* (PM) extracts, (A) neochlorogenic acid (B) chlorogenic acid, and (C) cryptochlorogenic acid. HPLC chromatograms of (D) MF, and (E) PM extracts.** mAU refers to the absorbance unit.

**Table 5.** Retention times and peak areas of Mume Fructus (MF) and *Prunus mume* (PM) extract (n=4).

| Name | | Retention time | Area | Area % | Height | Height % |
| --- | --- | --- | --- | --- | --- | --- |
| Neochlorogenic acid | | 10.82 | 3416.25 | 78.56 | 261.84 | 85.00 |
|  |  | 10.90 | 3347.41 | 78.29 | 256.00 | 85.69 |
|  |  | 10.96 | 3382.73 | 79.72 | 287.15 | 81.72 |
|  |  | 10.87 | 3293.04 | 79.31 | 279.02 | 82.90 |
| Chlorogenic acid | | 13.85 | 3699.57 | 76.60 | 274.59 | 79.78 |
|  |  | 13.90 | 3625.57 | 76.53 | 267.78 | 79.86 |
|  |  | 13.85 | 3076.21 | 75.58 | 227.75 | 76.33 |
|  |  | 13.78 | 3558.78 | 76.29 | 263.18 | 76.93 |
| Cryptochlorogenic acid | | 14.93 | 5581.36 | 90.09 | 366.02 | 92.60 |
|  |  | 14.84 | 5580.47 | 92.84 | 368.36 | 93.38 |
|  |  | 14.74 | 5854.79 | 90.71 | 384.17 | 92.93 |
|  |  | 14.63 | 5547.67 | 90.97 | 362.62 | 91.94 |
| Mume Fructus | Neochlorogenic acid | 10.75 | 4194.30 | 37.70 | 248.85 | 34.68 |
|  |  | 10.76 | 4196.36 | 37.67 | 253.10 | 35.20 |
|  |  | 10.77 | 4150.94 | 36.93 | 253.46 | 34.93 |
|  |  | 10.87 | 4041.00 | 37.77 | 313.25 | 39.30 |
|  | Chlorogenic acid | 13.82 | 1690.92 | 15.20 | 127.88 | 17.82 |
|  |  | 13.82 | 1696.46 | 15.23 | 128.58 | 17.88 |
|  |  | 13.83 | 1697.50 | 15.10 | 128.83 | 17.75 |
|  |  | 13.91 | 1665.17 | 15.56 | 126.96 | 15.93 |
|  | Cryptochlorogenic acid | 14.80 | 2516.18 | 22.62 | 147.30 | 20.53 |
|  |  | 14.80 | 2524.91 | 22.67 | 148.34 | 20.63 |
|  |  | 14.81 | 2526.57 | 22.48 | 148.34 | 20.44 |
|  |  | 14.95 | 2406.64 | 22.50 | 150.63 | 18.90 |
| *Prunus mume* | Neochlorogenic acid | 10.72 | 7363.15 | 39.01 | 530.13 | 37.32 |
|  |  | 10.72 | 7363.08 | 38.71 | 529.47 | 37.25 |
|  |  | 10.81 | 7288.23 | 37.15 | 503.99 | 36.23 |
|  |  | 10.72 | 7381.71 | 39.32 | 527.79 | 37.33 |
|  | Chlorogenic acid | 13.83 | 2906.94 | 15.40 | 219.76 | 15.47 |
|  |  | 13.83 | 2904.89 | 15.27 | 219.57 | 15.45 |
|  |  | 13.90 | 2851.50 | 14.54 | 217.62 | 15.64 |
|  |  | 13.83 | 2908.66 | 15.50 | 219.70 | 15.54 |
|  | Cryptochlorogenic acid | 14.936 | 1022.46 | 5.42 | 68.90 | 4.85 |
|  |  | 14.933 | 1022.52 | 5.38 | 69.50 | 4.89 |
|  |  | 14.955 | 942.33 | 5.02 | 69.72 | 4.94 |
|  |  | 14.927 | 941.90 | 5.02 | 69.48 | 4.91 |

Area, Area under the peak; Area %; Percentage of the peak area in the entire chromatogram; Height, Peak height; Height %, Percentage of the peak height in the entire chromatogram.

*15. Finally, the authors conclude that the differences that they observe are due to the higher concentration of 4-CQA in PM. To support this, an isolation of the 3 ingredients should be performed and these should be tested separately in primary cells.*

**Response**: First of all, we thank the reviewer for the valuable suggestion. We acknowledge that the reviewer’s idea is helpful in improving the quality of our work to a higher level. According to the reviewer's comment, we reviewed our work and reorganized the logical flow of the manuscript. Through experiments for comparisons between MF and PM extracts, it was confirmed that MF is effective in chondrocytes, and through analysis of the components of the extracts, it was confirmed that the MF extract contains a higher concentration of 4-CQA than the PM extract. In addition, through our investigation of the therapeutic mechanisms of MF in chondrocytes, it was revealed that MF inhibits inflammatory responses and expression of cartilage degradation-related markers through MAPK and NF-kB pathways, thereby showing efficacy in OA. Our study focused on verifying the efficacy of the MF extract itself. We are aware that we were unable to delve into the efficacy of the marker components, which is a limitation of this study, and we plan to conduct further studies to investigate the effect of marker components of MF on OA.

*16. A major limitation is that there is no support of these findings from in vivo experiments.*

**Response**: We agree with the reviewer’s view on the limitations of our study. We planned the present study based on the fact that there were no prior works investigating and verifying the efficacy of MF in cell experiments. We prepared this manuscript with the direction that through cell-level experiments, our study will be able to prompt more scientific research on MF. If we get to have the right opportunity available, we hope to conduct *in vivo* animal experiments on the efficacy of MF.

Reviewer #3:

*1. In my opinion, the paper is well structured and tells a story starting from a general concept and delves further and further into molecular details up to the detection of the molecule associated with the aforementioned anti-inflammatory power. However, the short chapter dedicated to MF/NSAIDs and pleiotropic effects has not been fully developed by the authors. I will attach my comments on this for more information.*

*Major points:*

*The authors correctly introduced the concept of possible pleiotropic effects of NSAIDs on chondrocytes. A publication by Chang et al. (Toxicology, 2006) analyzed the possible effects on proliferation and cell death of different groups of NSAIDs in cultured epiphyseal-articular chondrocytes of fetal rats. The authors concluded that non-selective NSAIDs cause reduced thymidine incorporation at dose-dependent concentrations, cell cycle arrest in the G_0_/G_1_ phase, and induction of both apoptosis and cell necrosis. Cyclooxygenase-2 (COX-2) selective NSAIDs accentuate these effects on chondrocytes in a less marked way and DFU, an analog of rofecoxib, has no effect on both cell proliferation and necrosis/apoptosis. Although the aforementioned research article shows the effects of NSAIDs on chondrocytes, a further experiment comparing NSAIDs and MF is important to understand whether MF may possess similar pleiotropic effects. A cytotoxicity test should be performed with NSAIDs (both non-selective and COX-2 selective NSAIDs since they have different power effects) at high concentrations as a positive control (DFU could be used as a negative control) and compared with MF at different doses to establish a possible non-involvement or even a benefit of MF versus cytotoxicity. I would suggest to add [3H]-thymidine incorporation assay, TUNEL stain (terminal deoxy-nucleotidyl transferase mediated dUTP nick end labeling stain), cell cycle kinetics by flow cytometry and caspase 3 activity assay to determine the effects of MF on cell proliferation, cell cycle and apoptosis/necrosis. I would apply the following experiments for both cultured rat condrocytes and macrophages to observe whether MF could have non-toxic effects on both chondrocytes and macrophages.*

**Response**: First of all, we thank the reviewer for the valuable comment. We acknowledge that the reviewer’s suggestion is helpful in improving the quality of this study to a higher level. Following the reviewer’s comment, we carefully reexamined the publication of Chang et al. (Toxicology, 2006) and realized that our sentence in the previous manuscript that described comparison with NSAIDs was not written properly. Because comparison with NSAIDs has no direct evidence based on experimental results, all relevant content in the manuscript was deleted or revised. This study aims to demonstrate that MF extract is effective for the treatment of OA through inhibition of inflammatory responses and cartilage degradation through MAPK and NF-kB pathways in chondrocytes, and therefore, there is no need to compare the efficacy of the MF extract with that of NSAIDs, for which there is no direct evidence. We address this issue in the following text added to the Discussion (Page 28, lines 557-566).

OA is a common joint disease with a steadily increasing prevalence that leads to chronic pain and impaired joint function and poses a global social burden [33, 34]. OA is a form of degenerative arthritis that affects the joints of the knees, hips, shoulders, hands, and feet and induces articular cartilage destruction and thinning [1]. Non-surgical modalities for the treatment of OA include medications such as hyaluronic acid, nonsteroidal anti-inflammatory drugs, and acetaminophen, while surgical treatment options include total knee arthroplasty, osteotomy, and unicompartmental knee arthroplasty [35]. However, complications, such as blood pressure problems and infections, have been reported following some of these treatments [36]. Therefore, plant extracts with joint protective effects and few side effects have been prioritized in the search for an alternative treatment for OA [37-39].

*2. In figure 4, the authors performed a western blot analysis to observe the role of IL-1β signaling in inflammation. It would be interesting to also introduce known inhibitors (U0126 for ERK1/2, SB239063 for p38 and SP600125 for JNK) in order to compare the inhibitory effect with MF.*

**Response**: First, we thank the reviewer for the valuable suggestions regarding our experiments on the mechanism involving the MAPK signaling pathways. We conducted the study based on the view that verifying the efficacy of *MF* in inhibiting IL1β-induced inflammatory markers or cartilage degradation in rat chondrocytes through experiments was the most appropriate method of evaluating the efficacy of MF. The main objective of our research was to explore the function of IL-1β signaling in inflammatory processes as it pertains to our study. Although existing inhibitors might offer insightful data regarding the implicated pathways, we deliberately concentrated on the innate reaction to MF, avoiding the influence of external inhibitors. By adopting this method, we were able to scrutinize the inherent signaling pathways and the natural effects of MF on chondrocytes.

*3. In figure 6, an immunofluorescence analysis of rat chondrocytes shows that the intensity of PM is comparable to the IL-1β group, suggesting no type of modulation of IL-1β signaling by PM. In addition to immunofluorescence, the authors could add a western blot with relative expression analysis of p-ERK/total ERK, p-p38/total p38 and p-JNK/total JNK to confirm at a biochemical level the absence of signaling modulation IL-1β versus MF (always consider cells treated with IL-1β only as a positive control).*

**Response**: First of all, we thank the reviewer for the valuable suggestion. We acknowledge that the reviewer’s idea is helpful in improving the quality of our work to a higher level. We reorganized the logical flow of the manuscript. Through the experiments for comparisons between MF and PM extracts, it was confirmed that MF is effective in chondrocytes, and through analysis of the components of the extracts, it was confirmed that the MF extract contains a higher concentration of 4-CQA than the PM extract. In addition, through our investigation of the therapeutic mechanisms of MF in chondrocytes, it was revealed that MF inhibits inflammatory responses and expression of cartilage degradation-related markers through MAPK and NF-kB pathways, thereby showing efficacy in OA. Please consider that our study focused on verifying the efficacy of the MF rather than on comparative evaluation between MF and PM

*4. Figure 7 of the article by Park and coworker shows an HPLC analysis between MF and PM with the aim of dissecting the profile of the chemical composition of the latter in order to understand why MF has an effect on the signaling dictated by IL-1β , while this is not the case for PM. The authors observed higher 4-CQA abundance in MF compared to PM, suggesting this molecule as responsible for the anti-inflammatory nature shown by MF. However, a molecular analysis of this molecule in the modulation of IL-1β-associated signaling remains to be determined/confirmed. Cryptochlorogenic acid powder can be purchased and can be applied in some experiments. Western blot analyses and immunofluorescence of rat chondrocytes treated with IL-1β, MF, PM and 4-CQA can be performed to re-observe the gene and protein expression of MMPs and collagen/aggrecan, as well as observe the phosphorylation levels of p-ERK, p-p38 and p-JNK, to confirm the key role of 4-CQA in inflammatory modulation in chondrocytes.*

**Response**: First of all, we thank the reviewer for the valuable suggestion. We acknowledge that the reviewer’s idea is helpful in improving the quality of our work to a higher level. According to the reviewer's comment, we reviewed our work and reorganized the logical flow of the manuscript. We address this issue in the following text added to the Results and Discussion (Page 21-32, lines 417-650).

Through experiments for comparisons between MF and PM extracts, it was confirmed that MF is effective in chondrocytes, and through analysis of the components of the extracts, it was confirmed that the MF extract contains a higher concentration of 4-CQA than the PM extract. In addition, through our investigation of the therapeutic mechanisms of MF in chondrocytes, it was revealed that MF inhibits inflammatory responses and expression of cartilage degradation-related markers through MAPK and NF-kB pathways, thereby showing efficacy in OA. Our study focused on verifying the efficacy of the MF extract itself rather than the efficacy of marker components of MF. We are aware that we were unable to delve into the efficacy of the marker components, which is a limitation of this study, and we plan to conduct further studies to investigate the effect of marker components of MF on OA. We added the same content in the text of the manuscript.

**Effect of *Mume Fructus* (MF) and *Prunus mume* (PM) on IL-1β-induced OA signaling in rat articular chondrocytes**

MF is processed from PM fruits via fumigation. Therefore, the effects of PM and MF were compared to confirm the effect of PM on the primary chondrocytes in rats. The cytotoxic effects of PM on rat chondrocytes were evaluated using a CCK-8 assay. The viability of PM- or IL-1β-treated chondrocytes remained unaffected at concentrations ranging from 0 to 100 μg/mL (Fig 1A). Compared with MF, PM did not affect IL-1β-induced OA signaling in the primary chondrocytes of rats (Fig 1B, C). These results were confirmed using immunofluorescence staining (Fig 2). MF has a more significant therapeutic effect on chondrocytes than PM.

**Fig 1. Comparison of the cytotoxicity and chondrogenic gene expression efficacy of Mume Fructus (MF) and *Prunus mume* (PM) on primary rat chondrocytes.** Rat articular chondrocytes were treated with or without MF and PM extracts (25, 50, and 100 μg/mL) and exposed to IL-1β (10 ng/ml) for 30 h. (A) Cell viability was assessed using the CCK-8 assay (n=4). (B, C) Metalloproteinase-3 (MMP3), metalloproteinase-13 (MMP13), a disintegrin and metalloproteinase with thrombospondin motifs 5 (ADAMTS5), collagen type II alpha 1 chain (COL2A1), and aggrecan (ACAN) mRNA levels were assessed using quantitative reverse transcription-PCR (n=5). Data are expressed as mean ± standard deviation (SD). One-way ANOVA was performed, followed by Dunnett’s multiple comparison test. *P < 0.05, **P < 0.01, ***P < 0.001 vs. IL-1β-treated group. #P < 0.05, ##P < 0.01, ###P < 0.001 vs. control group.

**Fig 2. *Prunus mume* (PM) had no effect on IL-1β-induced signaling in primary chondrocytes.** Expression of metalloproteinase-3 (MMP3) (A), a disintegrin and metalloproteinase with thrombospondin motifs 5 (ADAMTS5) (B), and collagen type II alpha 1 chain (COL2A1) (C) was evaluated using immunofluorescence staining (n=5). Scale bar = 25 μm. (D) The fluorescence intensity was quantified using image J. Data are expressed as mean ± standard deviation (SD). One-way ANOVA was performed, followed by Dunnett’s multiple comparison test. *P < 0.05, **P < 0.01, ***P < 0.001 vs. IL-1β-treated group. #P < 0.05, ##P < 0.01, ###P < 0.001 vs. control group.

**HPLC analysis identified the chemical composition of Mume Fructus (MF) and *Prunus mume* (PM) aqueous extracts**

To determine the differences in the chemical composition of MF and PM, which could be linked to their diverse effects on rat primary chondrocytes, phytochemical screening and analysis were performed using HPLC (Table 4).

**Table 4. Chemical profiles of the aqueous extracts of Mume Fructus (MF) and *Prunus mume* (PM)**

| No | Type of  phytochemical | Name | Formula | Aqueous extract |
| --- | --- | --- | --- | --- |
| 1 | Phenylpropanoids | Neochlorogenic acid | C_16_H_18_O_9_ | + |
| 2 |  | Chlorogenic acid | C_16_H_18_O_9_ | + |
| 3 |  | Cryptochlorogenic acid | C_16_H_18_O_9_ | + |
| 4 |  | 1,3-Di-caffeoylquinic acid | C_25_H_24_O_12_ | - |
| 5 |  | 3,5-Di-caffeoylquinic acid | C_25_H_24_O_12_ | - |
| 6 |  | 3,4-Di-caffeoylquinic acid | C_25_H_24_O_12_ | - |
| 7 |  | 3,5-Di-caffeoylquinic acid methyl ether | C_26_H_26_O_12_ | - |
| 8 |  | Caffeic acid | C_9_H_8_O_4_ | - |
| 9 |  | cis-*p*-Coumaric acid | C_9_H_8_O_3_ | - |
| 10 |  | trans-*p*-Coumaric acid | C_9_H_8_O_3_ | - |
| 11 |  | *p*-Coumaric acid | C_9_H_8_O_3_ | - |
| 12 |  | Ferulic acid | C_10_H_10_O_4_ | - |
| 13 |  | Ferulic acid - cis/trans mixture | C_10_H_10_O_4_ | - |
| 14 | Coumarins | Scopolin | C_16_H_18_O_9_ | - |
| 15 |  | Scopoletin | C_10_H_8_O_4_ | - |
| 16 | Flavonoids | Rutin | C_27_H_30_O_16_ | - |
| 17 |  | Isoquercetin | C_21_H_20_O_12_ | - |
| 18 |  | Apigenin-7-glucoside | C_21_H_20_O_10_ | - |
| 19 |  | Neoliquiritin | C_21_H_22_O_9_ | - |
| 20 |  | Catechin | C_15_H_14_O_6_ | - |
| 21 | Phenols | Gallic acid | C_7_H_6_O_5_ | - |
| 22 |  | 4-Hydroxybenzoic acid | C_7_H_6_O_3_ | - |
| 23 |  | 2,6-Dihydroxybenzoic acid | C_7_H_6_O_4_ | - |
| 24 | Sugar | 5-Hydroxymethyl-2-furaldehyde | C_6_H_6_O_3_ | - |

HPLC chromatograms showed peaks of neochlorogenic acid (Fig 3A), chlorogenic acid (Fig 3B), and cryptochlorogenic acid (4-CQA, Fig 3C) in both MF and PM extracts. Unlike the peak area distribution of the PM extract, the peak area of 4-CQA in the MF extract was more than 4 times larger than that in the PM extract; the peak area ratio in the MF extract was 22.62% whereas that in the PM extract was 5.42%, indicating a marked difference between the two peak areas. These results suggest that the 4-CQA component in MF inhibits OA signaling in the primary chondrocytes of rats. These results suggest that the 4-CQA component in MF inhibits OA signaling in the primary chondrocytes of rats.

**Fig 3.** **Chemical structure of the three main ingredients of Mume Fructus (MF) and *Prunus mume* (PM) extracts, (A) neochlorogenic acid (B) chlorogenic acid, and (C) cryptochlorogenic acid. HPLC chromatograms of (D) MF, and (E) PM extracts.** mAU refers to the absorbance unit.

**Table 5.** Retention times and peak areas of Mume Fructus (MF) and *Prunus mume* (PM) extract (n=4).

| Name | | Retention time | Area | Area % | Height | Height % |
| --- | --- | --- | --- | --- | --- | --- |
| Neochlorogenic acid | | 10.82 | 3416.25 | 78.56 | 261.84 | 85.00 |
|  |  | 10.90 | 3347.41 | 78.29 | 256.00 | 85.69 |
|  |  | 10.96 | 3382.73 | 79.72 | 287.15 | 81.72 |
|  |  | 10.87 | 3293.04 | 79.31 | 279.02 | 82.90 |
| Chlorogenic acid | | 13.85 | 3699.57 | 76.60 | 274.59 | 79.78 |
|  |  | 13.90 | 3625.57 | 76.53 | 267.78 | 79.86 |
|  |  | 13.85 | 3076.21 | 75.58 | 227.75 | 76.33 |
|  |  | 13.78 | 3558.78 | 76.29 | 263.18 | 76.93 |
| Cryptochlorogenic acid | | 14.93 | 5581.36 | 90.09 | 366.02 | 92.60 |
|  |  | 14.84 | 5580.47 | 92.84 | 368.36 | 93.38 |
|  |  | 14.74 | 5854.79 | 90.71 | 384.17 | 92.93 |
|  |  | 14.63 | 5547.67 | 90.97 | 362.62 | 91.94 |
| Mume Fructus | Neochlorogenic acid | 10.75 | 4194.30 | 37.70 | 248.85 | 34.68 |
|  |  | 10.76 | 4196.36 | 37.67 | 253.10 | 35.20 |
|  |  | 10.77 | 4150.94 | 36.93 | 253.46 | 34.93 |
|  |  | 10.87 | 4041.00 | 37.77 | 313.25 | 39.30 |
|  | Chlorogenic acid | 13.82 | 1690.92 | 15.20 | 127.88 | 17.82 |
|  |  | 13.82 | 1696.46 | 15.23 | 128.58 | 17.88 |
|  |  | 13.83 | 1697.50 | 15.10 | 128.83 | 17.75 |
|  |  | 13.91 | 1665.17 | 15.56 | 126.96 | 15.93 |
|  | Cryptochlorogenic acid | 14.80 | 2516.18 | 22.62 | 147.30 | 20.53 |
|  |  | 14.80 | 2524.91 | 22.67 | 148.34 | 20.63 |
|  |  | 14.81 | 2526.57 | 22.48 | 148.34 | 20.44 |
|  |  | 14.95 | 2406.64 | 22.50 | 150.63 | 18.90 |
| *Prunus mume* | Neochlorogenic acid | 10.72 | 7363.15 | 39.01 | 530.13 | 37.32 |
|  |  | 10.72 | 7363.08 | 38.71 | 529.47 | 37.25 |
|  |  | 10.81 | 7288.23 | 37.15 | 503.99 | 36.23 |
|  |  | 10.72 | 7381.71 | 39.32 | 527.79 | 37.33 |
|  | Chlorogenic acid | 13.83 | 2906.94 | 15.40 | 219.76 | 15.47 |
|  |  | 13.83 | 2904.89 | 15.27 | 219.57 | 15.45 |
|  |  | 13.90 | 2851.50 | 14.54 | 217.62 | 15.64 |
|  |  | 13.83 | 2908.66 | 15.50 | 219.70 | 15.54 |
|  | Cryptochlorogenic acid | 14.936 | 1022.46 | 5.42 | 68.90 | 4.85 |
|  |  | 14.933 | 1022.52 | 5.38 | 69.50 | 4.89 |
|  |  | 14.955 | 942.33 | 5.02 | 69.72 | 4.94 |
|  |  | 14.927 | 941.90 | 5.02 | 69.48 | 4.91 |

Area, area under the peak; Area %; percentage of the peak area in the entire chromatogram; Height, peak height; Height %, percentage of the peak height in the entire chromatogram.

**Mume Fructus (MF) treatment suppresses IL-1β-induced OA signaling in rat articular chondrocytes**

The CCK-8 assay was used to confirm the cytotoxic effects of MF on rat chondrocytes. The viability of MF-treated rat chondrocytes remained unaffected at MF concentrations ranging from 0 to 100 μg/mL, and the survival rate was dose-dependent in IL-1β-treated rat chondrocytes (Fig 1A). IL-1β is known to trigger the expression of various extracellular proteolytic enzymes in chondrocytes, such as *MMP3, MMP13,* and *ADAMTS5* [13, 14]. To elucidate the effect of MF extract on ECM synthesis, we examined IL-1β-induced ECM degradation in rat articular chondrocytes treated with multiple doses of MF extract for 30 h. qRT-PCR analysis revealed that MF extract inhibited *MMP3, MMP13*, and *ADAMTS5* expression (Fig 1B). These results were confirmed using western blotting (Fig 4A). Furthermore, as *Col2a1* is a component of the cartilage matrix, its degradation and reduction may initiate and promote OA progression [15]. MF treatment restored the IL-1β-induced decrease in *Col2a1* mRNA levels (Fig 1C). Moreover, western blot analysis revealed that MF treatment increased *COL2A1* expression (Fig 4B). The results were confirmed using immunofluorescence staining (Fig 5). Our findings suggest that the MF extract reduced the levels of OA markers (*MMP3, MMP13*, and *ADAMTS5*) and prevented the suppression of *COL2A1* expression.

**Fig 4. Effect of Mume Fructus (MF) on the protein levels of metalloproteinase-3 (MMP3), metalloproteinase-13 (MMP13), a disintegrin and metalloproteinase with thrombospondin motifs 5 (ADAMTS5), and collagen type II alpha 1 chain (COL2A1) in rat articular chondrocytes.** Rat articular chondrocytes were treated with or without MF extract (25, 50, and 100 μg/mL) and exposed to IL-1β (10 ng/mL) for 30 h. (A) MMP3, MMP13, ADAMTS5, and (B) COL2A1 protein expression was assessed using western blot analysis (n=4). Data are expressed as mean ± standard deviation (SD). One-way ANOVA was performed followed by Dunnett’s multiple comparisons test. *P < 0.05, **P < 0.01, ***P < 0.001 vs. IL-1β-treated group. #P < 0.05, ##P < 0.01, ###P < 0.001 vs. control group.

**Fig 5. Effects of Mume Fructus (MF) on IL-1β-induced signaling in primary chondrocytes. (A-C)** The expression of matrix metalloproteinase (MMP) 3 (A), a disintegrin and metalloproteinase with thrombospondin motifs 5 (ADAMTS5) (B), and collagen type II alpha 1 chain (COL2A1) (C) was evaluated using immunofluorescence staining (n=5). Scale bar = 25 μm. (D) Fluorescence intensity was quantified using Image J. Data are expressed as mean ± standard deviation (SD). One-way ANOVA was performed, followed by Dunnett’s multiple comparisons test. *P < 0.05, **P < 0.01, ***P < 0.001 vs. IL-1β-treated group. #P < 0.05, ##P < 0.01, ###P < 0.001 vs. control group.

**Mume Fructus (MF) treatment inhibits LPS-induced inflammation in macrophages**

The CCK-8 assay was used to examine the cytotoxic effects of MF on macrophages. The viability of MF- or LPS-treated RAW 264.7 cells was not affected at concentrations of MF ranging from 0 to 100 μg/mL (Fig 6A). LPS can activate macrophages to produce various pro-inflammatory cytokines, such as TNF-α and IL-6, and other inflammatory mediators, including NO [31, 32]. To assess the effect of MF on NO production in RAW 264.7 cells, we examined the inflammation induced by LPS and multiple doses of MF extract for over 24 h. MF treatment inhibited NO production in RAW 264.7 cells in a dose-dependent manner with and without LPS stimulation (Fig 6B). Moreover, TNF-α, IL-6, and IL-1β levels significantly increased in LPS-treated cells and decreased in MF-treated cells (Fig 6C).

**Fig 6. Effects of Mume Fructus (MF) on lipopolysaccharide (LPS)-induced inflammatory responses in RAW 264.7 cells.** RAW 264.7 cells were treated with or without MF extract (25, 50, and 100 μg/mL) and exposed to LPS (1 μg/mL) for 24 h. (A) Cell viability was assessed using the CCK-8 assay (n=4). (B) Nitric oxide (NO) production was determined using an NO assay (n=4). (C) mRNA levels of tumor necrosis factor-alpha (TNF-α), interleukin 6 (IL-6), and interleukin 1 beta (IL-1β) were detected via quantitative reverse transcription-PCR (n=4). Data are expressed as mean ± standard deviation (SD). One-way ANOVA was performed followed by Dunnett’s multiple comparison test. *P < 0.05, **P < 0.01, ***P < 0.001 vs. LPS-treated group. #P < 0.05, ##P < 0.01, ###P < 0.001 vs. control group.

**Mume Fructus (MF) treatment inhibits IL-1β-induced inflammatory signaling in rat chondrocytes**

Previous studies have reported that IL-1β-induced MAPK and NF-kB activation promotes MMP overproduction, thereby causing cartilage degradation [12, 17]. To confirm the mechanism underlying the effects of MF on rat chondrocytes, the MAPK signaling pathways were assessed using western blot analysis. The p-ERK, p-JNK, and p-p38 levels were significantly increased in IL-1β-stimulated cells and were decreased in MF-treated cells (Fig 7A). Moreover, IL-1β-activated p-p65 expression was inhibited by MF treatment (Fig 7B). Additionally, IL-1β induced the production of various pro-inflammatory cytokines and other inflammatory mediators, including COX-2. MF treatment also inhibited COX-2 (Fig 7C), TNF-α, IL-6, and IL-1β (Fig 7D) expression in rat articular chondrocytes. These results suggest that MF suppressed OA inflammation through MAPK and NF-kB signaling, consequently suppressing the expression of OA markers (*MMP3, MMP13*, and *ADAMTS5*) and restoring that of regeneration markers (such as *COL2A1*).

**Fig 7. Inhibition of inflammatory response upon Mume Fructus (MF) treatment via mitogen-activated protein kinase–nuclear factor-kappa B (MAPK–NF-κB) signaling in rat articular chondrocytes.** Rat articular chondrocytes were treated with or without MF extract (25, 50, and 100 μg/mL) and exposed to IL-1β (10 ng/mL) for (A) 30 min, (B) 1 h, and (C, D) 30 h (n=4). (A) Protein expression of phohphorylated extracellular signal-regulated kinase (p-ERK), phohphorylated c-Jun N-terminal kinase (p-JNK), and phohphorylated P38 (p-p38) assessed via western blot analysis. (B) Protein expression of phohphorylated P65 (p-p65) assessed via western blot analysis. (C) Protein expression of cyclooxygenase-2 (COX-2) assessed via western blot analysis. (D) tumor necrosis factor-alpha (TNF-α), interleukin 6 (IL-6), and interleukin 1 beta (IL-1β) mRNA levels were detected using quantitative reverse transcription-PCR. Data are expressed as mean ± standard deviation (SD). One-way ANOVA was performed followed by Dunnett’s multiple comparisons test. *P < 0.05, **P < 0.01, ***P < 0.001 vs. IL-1β-treated group. #P < 0.05, ##P < 0.01, ###P < 0.001 vs. control group.

**Discussion**

OA is a common joint disease with a steadily increasing prevalence that leads to chronic pain and impaired joint function and poses a global social burden [33, 34]. OA is a form of degenerative arthritis that affects the joints of the knees, hips, shoulders, hands, and feet and induces articular cartilage destruction and thinning [1]. Non-surgical modalities for the treatment of OA include medications such as hyaluronic acid, nonsteroidal anti-inflammatory drugs, and acetaminophen, while surgical treatment options include total knee arthroplasty, osteotomy, and unicompartmental knee arthroplasty [35]. However, complications, such as blood pressure problems and infections, have been reported following some of these treatments [36]. Therefore, plant extracts with joint protective effects and few side effects have been prioritized in the search for an alternative treatment for OA [37-39]. One candidate is MF, a traditional herbal product with anti-inflammatory, antioxidant, and abdominal pain-reducing effects [21, 26]; however, its effect on OA remains unknown. PM, a herbal medicinal plant commonly used in traditional Korean medicine and folk remedies, is the fruit of the Chinese plum (*Prunus mume* Sieb. et Zucc.). It has been reported to exhibit antimicrobial activity [40], and is effective against gastric secretion in rats [41] and diabetes [42]. PM has different names and uses depending on the time of harvest and processing method, and it is generally classified as follows: Cheongmae is a green fruit with hard pulp and a strong sour and astringent taste; Cheongmae in steamed and dried state is called Geummae; Cheongmae pickled in brine and sun-dried is called Baekmae; Cheongmae with its pericarp removed and blackened to charcoal is called Omae (MF); and the yellow fruit with the ripe, fragrant smell is called Hwangmae [43]. MF is processed by removing the pericarp and pits of near-mature Cheongmae picked from mid-June to early July, dried, and steam baked to black in a straw fire. MF is widely used as a medicinal herb in traditional Korean and Chinese medicine [44]. Previous studies on the effects of MF have reported its antioxidant activity [45], antimicrobial effects against bacteria causing food poisoning/gastroenteritis [46], antitumor effects [47], and hypoglycemic effects [48]. However, few studies have specifically investigated the potential of using MF in the treatment of OA.

PM is used to treat various symptoms, including fatigue, cough, headaches, constipation, food poisoning, and stomach disorders [49]. Pharmacologically, PM has been studied as an antidiabetic [50], hepatoprotective [51], antitumour [52], anti-inflammatory [53, 54], antibacterial [55], and anticancer agent [50]. We predicted that PM may also have an anti-inflammatory effect, thereby exerting an inhibitory effect on cartilage destruction due to an inflammatory response. However, PM, the raw material of MF, did not affect OA signaling in the chondrocytes. Assuming that the differential responses could be ascribed to different chemical compositions, we conducted phytochemical analysis of MF and PM via HPLC. Our results revealed the presence of three main compounds, with the 4-CQA content differing between MF and PM. 4-CQA is a phenolic acid and unique isomer of chlorogenic acid [56]. Its pharmacological effects include improvement in the blood glucose levels, iron content, and lipid peroxide accumulation via activation of the system XC/glutathione peroxidase 4/factor-erythroid factor 2 system in patients with diabetes [57]. Additionally, 4-CQA attenuates LPS-induced inflammatory responses and oxidative stress and ameliorates myocardial hypertrophy through a hypoxia-inducible factor 1 subunit alpha-related pathway [57, 58]. The anti-inflammatory effects of 4-CQA may be attributed to its ability to inhibit OA through MF-mediated inflammatory signaling regulation.

IL-1β induces the expression of several inflammatory mediators, such as IL-6, TNF-α, leukaemia inhibitory factor IL-6 family cytokine, prostaglandin E2, NO, COX-2, and inducible NO synthase, and various catabolic processes; it further contributes to synovial inflammation that degrades the cartilage. This process of IL-1β-induced cartilage destruction activates all three MAPKs (ERK, p38, and JNK) and the NF-κB signal cascade pathway [19]. These intracellular signaling pathways induce OA owing to their participation in ECM degradation homeostasis, resulting in increased *MMP3*, *MMP13*, and *ADAMTS5* levels and reduced *COL2A1* levels [59, 60].

The pathophysiology of OA is characterized by the breakdown of the ECM of articular cartilage by proteinases, whose expression is upregulated by inflammatory stimuli [61]. These inflammatory responses occur in chondrocytes and synovial macrophages [62]. Among the pathways involved in inflammatory responses, MAPKs and NF-кB are well known to play key roles [63]. Therefore, we examined and confirmed the anti-inflammatory effects of MF through gene expression of TNF-α, IL-6, and IL-1β in RAW264.7 cells and also in chondrocytes.

Our results verified the effects of MF treatment in inhibiting MMP3/13 and increasing COL2A1 expression, which were combined to demonstrate the efficacy of MF treatment in OA signaling inhibition (Fig 8). Additionally, MF treatment reduced TNF-α, IL-6, and IL-1β levels in a dose-dependent manner in macrophages and chondrocytes. These OA signaling inhibitions and anti-inflammatory effects were induced by MF, which regulated all three MAPKs and NF-kB signaling. Moreover, MF treatment reduced the levels of COX-2, which is another inflammatory mediator.

**Fig 8. Mechanistic overview of promoting extracellular matrix regeneration and inhibiting degradation in IL-1β-induced chondrocytes by Mume Fructus (MF)**. MF effectively suppressed MAPK/NF-kB signalling after IL-1β-induced damage by significantly suppressing the inflammatory response and consequently promoting extracellular matrix regeneration and inhibiting degradation.

Notably, the increase in the COL2A1 expression in the MF treatment group was considerably higher than that in the control group. Collagen is the most abundant protein in mammals [64]. In humans, different types of collagens are present in the connective tissues such as discs, cartilage, tendons, and ligaments of diverse organs such as bones and joints [65]. COL2A1, which comprises discs and cartilages, is degraded by MMP3 and MMP13 [15]. A recent study indicated that the degradation of COL2A1 and a decrease in its level might initiate and promote OA [15]; moreover, the study suggested that collagen degradation played a central role in the pathogenesis of OA. Therefore, the upregulation of COL2A1 expression induced by MF in cartilage may be an OA treatment strategy. Additionally, MF could induce COL2A1 expression and inhibit MMP3 expression in discs during the treatment of herniated discs using Jaseng Woongayoungsin-hwan; however, further research is warranted in this regard.

Our study has a limitation. As we focused on the efficacy of MF extract in chondrocytes, we were able to analyze the marker components of MF but not to the extent of component-specific effects. Further studies are needed to determine the specific effects of 4-CQA on chondrocytes and OA pathogenesis to determine the specific effects of 4-CQA on chondrocytes and OA pathogenesis.

**Conclusions**

The current study revealed that MF treatment reduces OA pathogenesis by inhibiting inflammation via MAPK–NF-κB signaling and induces COL2A1 expression. Thus, MF could be a potential therapeutic agent for OA, promoting ECM regeneration and inhibiting its degradation.

**Minor points**

*1. Discussion section (lines 331-337)*. I would reccomend to add the following citation :

Chang JK, Wu SC, Wang GJ, Cho MH, Ho ML. Effects of non-steroidal anti-inflammatory drugs on cell proliferation and death in cultured epiphyseal-articular chondrocytes of fetal rats. Toxicology. 2006 Dec 7;228(2-3):111-23. doi: 10.1016/j.tox.2006.08.028. Epub 2006 Aug 30. PMID: 17045721.

**Response**: First of all, we thank the reviewer for the valuable comment. We acknowledge that the reviewer’s suggestion is helpful in improving the quality of this study to a higher level. Following the reviewer’s comment, we carefully reexamined the publication of Chang et al. (Toxicology, 2006) and realized that our sentence in the previous manuscript that described comparison with NSAIDs was not written properly. Because comparison with NSAIDs has no direct evidence based on experimental results, all relevant content in the manuscript was deleted or revised. This study aims to demonstrate that MF extract is effective for the treatment of OA through inhibition of inflammatory responses and cartilage degradation through MAPK and NF-kB pathways in chondrocytes, and therefore, there is no need to compare the efficacy of the MF extract with that of NSAIDs, for which there is no direct evidence. We address this issue in the following text added to the Discussion (Page 28, lines 557-566).

OA is a common joint disease with a steadily increasing prevalence that leads to chronic pain and impaired joint function and poses a global social burden [33, 34]. OA is a form of degenerative arthritis that affects the joints of the knees, hips, shoulders, hands, and feet and induces articular cartilage destruction and thinning [1]. Non-surgical modalities for the treatment of OA include medications such as hyaluronic acid, nonsteroidal anti-inflammatory drugs, and acetaminophen, while surgical treatment options include total knee arthroplasty, osteotomy, and unicompartmental knee arthroplasty [35]. However, complications, such as blood pressure problems and infections, have been reported following some of these treatments [36]. Therefore, plant extracts with joint protective effects and few side effects have been prioritized in the search for an alternative treatment for OA [37-39].

*2. Figure section*. Improve figures quality. I opened the document several times and the file lost its initial resolution. I would suggest saving the images in TIFF format and mounting them in Adobe® Photoshop/Illustrator to preserve resolution quality.

**Response**: We thank you for your comment. All Figures were saved in TIFF format, the resolution was also improved to 300DPI and uploaded accordingly. If you still have problems with the resolution of Figures, please contact us at any time and we will send you the original file.

*3. Figure section*. Add concentration units in each graph.

**Response**: We apologize for making it difficult for the reviewers to understand due to omitted units from the figure. We have added units to all figures for the necessary modifications.

**Fig 1. Comparison of the cytotoxicity and chondrogenic gene expression efficacy of Mume Fructus (MF) and *Prunus mume* (PM) on primary rat chondrocytes.**

**Fig 2. *Prunus mume* (PM) had no effect on IL-1β-induced signaling in primary chondrocytes.**

**Fig 3.** **Chemical structure of the three main ingredients of Mume Fructus (MF) and *Prunus mume* (PM) extracts, (A) neochlorogenic acid (B) chlorogenic acid, and (C) cryptochlorogenic acid. HPLC chromatograms of (D) MF, and (E) PM extracts.**

**Fig 4. Effect of Mume Fructus (MF) on the protein levels of metalloproteinase-3 (MMP3), metalloproteinase-13 (MMP13), a disintegrin and metalloproteinase with thrombospondin motifs 5 (ADAMTS5), and collagen type II alpha 1 chain (COL2A1) in rat articular chondrocytes.**

**Fig 5. Effects of Mume fructus (MF) on IL-1β-induced signalling in primary chondrocytes.**

**Fig 6. Effects of Mume Fructus (MF) on lipopolysaccharide (LPS)-induced inflammatory responses in RAW 264.7 cells.**

**Fig 7. Inhibition of inflammatory response upon Mume Fructus (MF) treatment via mitogen-activated protein kinase–nuclear factor-kappa B (MAPK–NF-κB) signaling in rat articular chondrocytes.**

*4. Figure section.* Quantitative analyzes of Western Blots show no error bars. I assume the experiments were not repeated. Repeat the experiments in order to obtain the minimum number of biological replicates so that a given significance can be obtained (if a significant finding is observed).

**Response**: We thank you for your comment. For western blot analysis, we rechecked about replicate measurements, and revised the legends of the Figures and the graphs used. We address this issue in the following text added to the Results (Page 25, lines 490-498; Page 28, lines 542-555).

**Fig 4. Effect of Mume Fructus (MF) on the protein levels of metalloproteinase-3 (MMP3), metalloproteinase-13 (MMP13), a disintegrin and metalloproteinase with thrombospondin motifs 5 (ADAMTS5), and collagen type II alpha 1 chain (COL2A1) in rat articular chondrocytes.** Rat articular chondrocytes were treated with or without MF extract (25, 50, and 100 μg/mL) and exposed to IL-1β (10 ng/mL) for 30 h. (A) MMP3, MMP13, ADAMTS5, and (B) COL2A1 protein expression was assessed using western blot analysis (n=4). Data are expressed as mean ± standard deviation (SD). One-way ANOVA was performed followed by Dunnett’s multiple comparisons test. *P < 0.05, **P < 0.01, ***P < 0.001 vs. IL-1β-treated group. #P < 0.05, ##P < 0.01, ###P < 0.001 vs. control group.

**Fig 7. Inhibition of inflammatory response upon Mume Fructus (MF) treatment via mitogen-activated protein kinase–nuclear factor-kappa B (MAPK–NF-κB) signaling in rat articular chondrocytes.** Rat articular chondrocytes were treated with or without MF extract (25, 50, and 100 μg/mL) and exposed to IL-1β (10 ng/mL) for (A) 30 min, (B) 1 h, and (C, D) 30 h (n=4). (A) Protein expression of phohphorylated extracellular signal-regulated kinase (p-ERK), phohphorylated c-Jun N-terminal kinase (p-JNK), and phohphorylated P38 (p-p38) assessed via western blot analysis. (B) Protein expression of phohphorylated P65 (p-p65) assessed via western blot analysis. (C) Protein expression of cyclooxygenase-2 (COX-2) assessed via western blot analysis. (D) tumor necrosis factor-alpha (TNF-α), interleukin 6 (IL-6), and interleukin 1 beta (IL-1β) mRNA levels were detected using quantitative reverse transcription-PCR. Data are expressed as mean ± standard deviation (SD). One-way ANOVA was performed followed by Dunnett’s multiple comparisons test. *P < 0.05, **P < 0.01, ***P < 0.001 vs. IL-1β-treated group. #P < 0.05, ##P < 0.01, ###P < 0.001 vs. control group

*5. Figure 7*. The 3 molecules taken into account in the HPLC peak analysis are shown. Molecules have letters A to C, peaks numbers from 1 to 3. It is true that it is specified in the text under image 7, but in my opinion it is easier to choose either numbers or letters to visually appreciate which molecule corresponds to which peak.

**Response**: We thank you for your comment. According to the reviewer's comment, the method of using numbers and letters for HPLC chromatograms was unified and revised to A, B, and C.

**Fig 3.** **Chemical structure of the three main ingredients of Mume Fructus (MF) and *Prunus mume* (PM) extracts, (A) neochlorogenic acid (B) chlorogenic acid, and (C) cryptochlorogenic acid. HPLC chromatograms of (D) MF, and (E) PM extracts.** mAU refers to the absorbance unit.

1.	Bae Y-K, Choe T-B. Antioxidant and cell activity using extracts of mume fructus. Korean Journal of Medicinal Crop Science. 2011; 19(5):388-94.

2.	Ku G, Kwon O, Roh S-S, Seo Y-B. Biological activity review of mume fructus and processed mume fructus. The Korea Journal of Herbology. 2016; 31(3):59-69.

3.	Seo C HH, Lee HY, Lee J, Jung D, Lee J, Shin HK. Simultaneous Analysis and Anti-allergic Effect of Mume Fructus. Korean Journal of Pharmacognosy 2012; 43(4):279–85.

4.	Shen H, Cheng T, Qiao C, Su Z, Li C. Antitumor effect in vitro and immuno-response in vivo of fructus Mume. Zhongguo Zhong yao za zhi= Zhongguo Zhongyao Zazhi= China Journal of Chinese Materia Medica. 1995; 20(6):365-8, inside back cover.

5.	Tantowi NCA, Mohamed S, Hussin P. Effect of ficus deltoidea, a medicinal plant, on cartilage protection in cartilage explant and postmenopausal rat models of osteoarthritis. Osteoarthritis and Cartilage. 2016; 24:S353-S4.

6.	Han SJ, Lee H, Nam J, Pan CH, Jeon J, Yang S. Schisandra extract ameliorates arthritis pathogenesis by suppressing the NF‐κB and MAPK signalling pathways. Journal of Cellular and Molecular Medicine. 2023.

7.	Kanwar JR, Samarasinghe RM, Kumar K, Arya R, Sharma S, Zhou S-F, et al. Cissus quadrangularis inhibits IL-1β induced inflammatory responses on chondrocytes and alleviates bone deterioration in osteotomized rats via p38 MAPK signaling. Drug design, development and therapy. 2015:2927-40.
