# Supplementary material for: Mume Fructus reduces interleukin-1 beta-induced cartilage degradation via MAPK downregulation in rat articular chondrocytes
Source: PLoS One. 2024 May 8;19(5):e0302906. doi: 10.1371/journal.pone.0302906 (PMC11078424; doi:10.1371/journal.pone.0302906)
Supplement: Attachment — Submitted filename: Reviewer comments#PLOSONE.docx [file pone.0302906.s007.docx]

Dear PLOS ONE Editor

The primary goal of the manuscript titled "Mume Fructus reduces interleukin-1 beta-induced cartilage degradation via MAPK down regulation in rat articular chondrocytes" is to clarify the biological process by which a herbal remedy works to mitigate the damaging effects of IL1β on cartilage, using this well-established in vitro model of osteoarthritis.

To support this theory, the authors have looked into the expression of key ECM-destructive enzymes, inflammatory cytokines, and components of the MAPK signaling cascade. They also looked at how MF affected macrophage inflammation brought on by LPS. The findings indicate that MF treatment reduces inflammation in LPS-treated macrophages and decreases OA signaling in chondrocytes produced by IL-1β.

They suggested that Mume Fructus (MF) could be a promising therapeutic agent for OA based on their hypothesis that MF treatment lowers OA pathogenesis by decreasing inflammation via MAPK–NF-κB signaling.

This assertion is corroborated by the western blotting technique results as well as qPCR analysis. After utilizing HPLC to examine the chemical content of MF, the researchers came to the conclusion that the 4-CQA component of MF is what inhibits OA signaling in chondrocytes.

Overall, the authors' results are well-supported by thorough research, and the manuscript itself has theoretical and structural significance. Nonetheless, the writers must address the following several issues:

1. The authors concluded that MF inhibited interleukin-1 beta-induced cartilage degradation via MAPK down regulation, citing references 17–19. They also mentioned that IL1β induces its destructive effects through activation of the MAPK signaling pathway. More proof, though, is required to support this. This theory might be validated by gene silencing certain MAPK signaling pathway components.
2. What software has been utilized for the analysis of immunofluorescence and western blot images?
3. Fig. 3 (B&C) are the same whether LPS treatment is applied or not. They are combinable.
4. Table 2 should include a description of the abbreviations for the phosphorylated forms of NF-ΚB, p65, ERK, JNK, and P38.
5. Verify the chromogram again in Fig. 7-D. There is no greater abundance of the 4-CQA peak in MF extract than in PM.
6. In Discussion, lines 346–348 *"The current study revealed that…… chondrocytes,"* but we are unable to draw the conclusion that the primary cause of the decrease in MMP3/13-ADAMTS-5 is the accumulation of COL2A as a result of MF treatment of chondrocytes. While over-expression of COL2A1 may be a useful treatment strategy for osteoarthritis, it is unable to decrease matrix metalloproteinase enzyme expression.
7. The authors found that the NFĸB and MAPK pathways are in charge of the OA cartilage destruction after studying the IL1β induced inflammatory signaling pathway in rat chondrocytes. Along these lines, a pertinent question concerning LPS-induced inflammation in macrophages emerges and needs to be addressed. Is there a comparable signaling pathway regarding the inflammation that LPS causes in macrophages?

Best regards

Davood Nasrabadi
